# Putting the Self in Self-Correction: Findings From the Loss-of-Confidence Project

**DOI:** 10.1177/1745691620964106

**Published:** 2021-03-01

**Authors:** Julia M. Rohrer, Warren Tierney, Eric L. Uhlmann, Lisa M. DeBruine, Tom Heyman, Benedict Jones, Stefan C. Schmukle, Raphael Silberzahn, Rebecca M. Willén, Rickard Carlsson, Richard E. Lucas, Julia Strand, Simine Vazire, Jessica K. Witt, Thomas R. Zentall, Christopher F. Chabris, Tal Yarkoni

**Affiliations:** 1 International Max Planck Research School on the Life Course, Max Planck Institute for Human Development, Berlin; 2 Department of Psychology, University of Leipzig; 3 Department of Organizational Behavior, INSEAD, Singapore; 4 Institute of Neuroscience and Psychology, University of Glasgow; 5 Laboratory of Experimental Psychology, KU Leuven; 6 Institute of Psychology, Leiden University; 7 Sussex Business School, University of Sussex; 8 Institute for Globally Distributed Open Research and Education (IGDORE); 9 Department of Psychology, Linnaeus University; 10 Department of Psychology, Michigan State University; 11 Department of Psychology, Carleton College; 12 Melbourne School of Psychological Sciences, University of Melbourne; 13 Department of Psychology, Colorado State University; 14 Department of Psychology, University of Kentucky; 15 Autism and Developmental Medicine Institute, Geisinger Health System, Danville, Pennsylvania; 16 Department of Psychology, University of Texas at Austin

**Keywords:** self-correction, knowledge accumulation, metascience, scientific falsification, incentive structure, scientific errors

## Abstract

Science is often perceived to be a self-correcting enterprise. In principle, the assessment of scientific claims is supposed to proceed in a cumulative fashion, with the reigning theories of the day progressively approximating truth more accurately over time. In practice, however, cumulative self-correction tends to proceed less efficiently than one might naively suppose. Far from evaluating new evidence dispassionately and infallibly, individual scientists often cling stubbornly to prior findings. Here we explore the dynamics of scientific self-correction at an individual rather than collective level. In 13 written statements, researchers from diverse branches of psychology share why and how they have lost confidence in one of their own published findings. We qualitatively characterize these disclosures and explore their implications. A cross-disciplinary survey suggests that such loss-of-confidence sentiments are surprisingly common among members of the broader scientific population yet rarely become part of the public record. We argue that removing barriers to self-correction at the individual level is imperative if the scientific community as a whole is to achieve the ideal of efficient self-correction.

Science is often hailed as a self-correcting enterprise. In the popular perception, scientific knowledge is cumulative and progressively approximates truth more accurately over time (Sismondo, 2010). However, the degree to which science is genuinely self-correcting is a matter of considerable debate. The truth may or may not be revealed eventually, but errors can persist for decades, corrections sometimes reflect lucky accidents rather than systematic investigation and can themselves be erroneous, and initial mistakes might give rise to subsequent errors before they get caught (Allchin, 2015). Furthermore, even in a self-correcting scientific system, it remains unclear how much of the knowledge base is credible at any given point in time (Ioannidis, 2012) given that the pace of scientific self-correction may be far from optimal.

Usually, self-correction is construed as an outcome of the activities of the scientific community as a whole (i.e., collective self-correction): Watchful reviewers and editors catch errors before studies get published, critical readers write commentaries when they spot flaws in somebody else’s reasoning, and replications by impartial groups of researchers allow the scientific community to update their beliefs about the likelihood that a scientific claim is true. Far less common are cases in which researchers publicly point out errors in their own studies and question conclusions they have drawn before (i.e., individual self-correction). The perceived unlikeliness of such an event is facetiously captured in Max Planck’s famous statement that new scientific truths become established not because their enemies see the light but because those enemies eventually die (Planck, 1948). However, even if individual self-correction is not necessary for a scientific community as a whole to be self-correcting in the long run (Mayo-Wilson et al., 2011), we argue that it can increase the overall efficiency of the self-corrective process and thus contribute to a more accurate scientific record.

## The Value of Individual Self-Correction

The authors of a study have privileged access to details about how the study was planned and conducted, how the data were processed or preprocessed, and which analyses were performed. Thus, the authors remain in a special position to identify or confirm a variety of procedural, theoretical, and methodological problems that are less visible to other researchers.^1^ Even when the relevant information can in principle be accessed from the outside, correction by the original authors might still be associated with considerably lower costs. For an externally instigated correction to take place, skeptical “outsiders” who were not involved in the research effort might have to carefully reconstruct methodological details from a scant methods section (for evidence that often authors’ assistance is required to reproduce analyses, see e.g., Chang & Li, 2018; Hardwicke et al., 2018), write persuasive e-mails to get the original authors to share the underlying data (often to no avail; Wicherts et al., 2011), recalculate statistics because reported values are not always accurate (e.g., Nuijten et al., 2016), or apply advanced statistical methods to assess evidence in the presence of distortions such as publication bias (Carter et al., 2019).

Eventually, external investigators might resort to an empirical replication study to clarify the matter. A replication study can be a very costly or even impossible endeavor. Certainly, it is inefficient when a simple self-corrective effort by the original authors might have sufficed. Widespread individual self-correction would obviously not eliminate the need for replication, but it would enable researchers to make better informed choices about whether and how to attempt replication—more than 30 million scientific articles have been published since 1965 (Pan et al., 2018), and limited research resources should not be expended mindlessly on attempts to replicate everything (see also Coles et al., 2018). In some cases, individual self-correction could render an empirical replication study unnecessary. In other cases, additionally disclosed information might render an empirical replication attempt even more interesting. And in any case, full information about the research process, including details that make the original authors doubt their claims, would help external investigators maximize the informativeness of their replication or follow-up study.

Finally, in many areas of science, scientific correction has become a sensitive issue often discussed with highly charged language (Bohannon, 2014). Self-correction could help defuse some of this conflict. A research culture in which individual self-corrections are the default reaction to errors or misinterpretations could raise awareness that mistakes are a routine part of science and help separate researchers’ identities from specific findings.

## The Loss-of-Confidence Project

To what extent does our research culture resemble the self-correcting ideal, and how can we facilitate such behavior? To address these questions and to gauge the potential impacts of individual self-corrections, we conducted the Loss-of-Confidence Project. The effort was born out of a discussion in the Facebook group PsychMAP following the online publication of Dana Carney’s statement “My Position on Power Poses” (Carney, 2016). Carney revealed new methodological details regarding one of her previous publications and stated that she no longer believed in the originally reported effects. Inspired by her open disclosure, we conducted a project consisting of two parts: an open call for loss-of-confidence statements and an anonymous online survey.

First, in our open call, we invited psychological researchers to submit statements describing findings that they had published and in which they had subsequently lost confidence.^2^ The idea behind the initiative was to help normalize and destigmatize individual self-correction while, hopefully, also rewarding authors for exposing themselves in this way with a publication. We invited authors in any area of psychology to contribute statements expressing a loss of confidence in previous findings, subject to the following requirements:

The study in question was an empirical report of a novel finding.
The submitting author has lost confidence in the primary/central result of the article.
The loss of confidence occurred primarily as a result of theoretical or methodological problems with the study design or data analysis.
The submitting author takes responsibility for the errors in question.

The goal was to restrict submissions to cases in which the stigma of disclosing a loss of confidence in previous findings would be particularly high; we therefore did not accept cases in which an author had lost faith in a previous finding for reasons that did not involve his or her own mistakes (e.g., because of a series of failed replications by other researchers).

Second, to understand whether the statements received in the first part of the project are outliers or reflect a broader phenomenon that goes largely unreported, we carried out an online survey and asked respondents about their experience with losses of confidence. The full list of questions asked can be found at https://osf.io/bv48h/. The link to the survey was posted on Facebook pages and mailing lists oriented toward scientists (PsychMAP, Psychological Methods Discussion Group, International Social Cognition Network, Society for Judgment and Decision Making (SJDM), SJDM mailing list) and further promoted on Twitter. Survey materials and anonymized data are made available on the project’s Open Science Framework repository (https://osf.io/bv48h).

## Results: Loss-of-Confidence Statements

The project was disseminated widely on social media (resulting in around 4,700 page views of the project website), and public commentary was overwhelmingly positive, highlighting how individual self-correction is aligned with perceived norms of scientific best practices. By the time we stopped the initial collection of submissions (December 2017–July 2018), we had received loss-of-confidence statements pertaining to six different studies. After posting a preprint of an earlier version of this manuscript, we reopened the collection of statements and received seven more submissions, some of them while finalizing the manuscript. Table 1 provides an overview of the statements we received.^3^

**Table 1.** Overview of the Loss-of-Confidence Statements

Authors	Title	Journal	JIF	Citations
Carlsson and Björklund (2010)	Implicit stereotype content: Mixed stereotypes can be measured with the implicit association test	*Social Psychology*	1.36	74
Chabris and Hamilton (1992)	Hemispheric specialization for skilled perceptual organization by chessmasters	*Neuropsychologia*	2.87	28
Fisher et al. (2015)	Women’s preference for attractive makeup tracks changes in their salivary testosterone	*Psychological Science*	4.90	9
Heyman et al. (2015)	The influence of working memory load on semantic priming	*Journal of Experimental Psychology: Learning, Memory and Cognition*	2.67	51
Lucas and Diener (2001)	Understanding extraverts’ enjoyment of social situations: The importance of pleasantness	*Journal of Personality and Social Psychology*	5.92	220
Schmukle et al. (2007)	Second to fourth digit ratios and the implicit gender self-concept	*Personality and Individual Differences*	2.00	20
Silberzahn and Uhlmann (2013)	It pays to be Herr Kaiser: Germans with noble-sounding surnames more often work as managers than as employees	*Psychological Science*	4.90	28
Smith and Zentall (2016)	Suboptimal choice in pigeons: Choice is primarily based on the value of the conditioned reinforcer rather than overall reinforcement rate	*Journal of Experimental Psychology: Animal Learning and Cognition*	2.03	64
Strand et al. (2018)	Talking points: A modulating circle reduces listening effort without improving speech recognition	*Psychonomic Bulletin & Review*	3.70	9
Vazire (2010)	Who knows what about a person? The self-other knowledge asymmetry (SOKA) model	*Journal of Personality and Social Psychology*	5.92	740
Willén and Strömwall (2012)	Offenders’ lies and truths: An evaluation of the supreme court of Sweden’s criteria for credibility assessment	*Psychology Crime & Law*	1.46	19
Witt and Proffitt (2008)	Action-specific influences on distance perception: A role for motor simulation	*Journal of Experimental Psychology: Human Perception and Performance*	2.94	252
Yarkoni et al. (2005)	Prefrontal brain activity predicts temporally extended decision-making behavior	*Journal of the Experimental Analysis of Behavior*	2.15	45

Note: JIF = 2018 journal impact factor according to InCites Journal Citation Reports. Citations are according to Google Scholar on April 27, 2019.

In the following, we list all statements in alphabetical order of the first author of the original study to which they pertain. Some of the statements have been abbreviated; the long versions are available at OSF (https://osf.io/bv48h/).

### Statement on Carlsson and Björklund (2010) by Rickard Carlsson

In this study, we developed a new way to measure mixed (in terms of warmth and competence) stereotypes with the help of the implicit association test (IAT). In two studies, respondents took two IATs, and results supported the predictions: Lawyers were implicitly stereotyped as competent (positive) and cold (negative) relative to preschool teachers. In retrospect, there are a number of issues with the reported findings. First, there was considerable flexibility in what counted as support for the theoretical predictions. In particular, the statistical analysis in Study 2 tested a different hypothesis than Study 1. This analysis was added after peer review Round 2 and thus was definitely not predicted a priori. Later, when trying to replicate the reported analysis from Study 1 on the data from Study 2, I found that only one of the two effects reported in Study 1 could be successfully replicated. Second, when we tried to establish the convergent and discriminant validity of the IATs by correlating them with explicit measures, we committed the fallacy of taking a nonsignificant effect in an underpowered test as evidence for the null hypothesis, which in this case implied discriminant validity. Third, in Study 1, participants actually took a third IAT that measured general attitudes toward the groups. This IAT was not disclosed in the manuscript and was highly correlated with both the competence and the warmth IAT. Hence, it would have complicated our narrative and undermined the claim that we had developed a completely new measure. Fourth, data from an undisclosed behavioral measure were collected but never entered into data set or analyzed because I made a judgment that it was invalid based on debriefing of the participants. In conclusion, in this 2010 article, I claimed to have developed a way to measure mixed stereotypes of warmth and competence with the IAT. I am no longer confident in this finding.

### Statement on Chabris and Hamilton (1992) by Christopher F. Chabris

This article reported a divided-visual-field (DVF) experiment showing that the skilled pattern recognition that chess masters perform when seeing a chess game situation was performed faster and more accurately when the stimuli were presented briefly in the left visual field, and thus first reached the right hemisphere of the brain, than when the stimuli were presented in the right field. The sample was large for a study of highly skilled performers (16 chess masters), but we analyzed the data in many different ways and reported the result that was most favorable. Most critically, we tried different rules for removing outlier trials and picked one that was uncommon but led to results consistent with our hypothesis. Nowadays, I would analyze these types of data using more justifiable rules and preregister the rules I was planning to use (among other things) to avoid this problem. For these reasons, I no longer think that the results provide sufficient support for the claims that the right hemisphere is more important than the left for chess expertise and for skilled visual pattern recognition. These claims may be true, but not because of our experiment.

Two other relevant things happened with this article. First, we submitted a manuscript describing two related experiments. We were asked to remove the original Experiment 1 because the *p* value for the critical hypothesis test was below .10 but not below .05. We complied with this request. We were also asked by one reviewer to run approximately 10 additional analyses of the data. We did not comply with this—instead, we wrote to the editor and explained that doing so many different analyses of the same data set would invalidate the *p* values. The editor agreed. This is evidence that the dangers of multiple testing were not exactly unknown as far back as the early 1990s. The sacrificed Experiment 1 became a chapter of my PhD thesis. I tried to replicate it several years later, but I could not recruit enough chess master participants. Having also lost some faith in the DVF methodology, I put that data in the “file drawer” for good.

### Statement on Fisher et al. (2015) by Ben Jones and Lisa M. DeBruine

The article reported that women’s preferences for wearing makeup that was rated by other people as being particularly attractive were stronger in test sessions in which salivary testosterone was high than in test sessions in which salivary testosterone was relatively low. Not long after publication, we were contacted by a colleague who had planned to use the open data and analysis code from our article for a workshop on mixed effect models. They expressed some concerns about how our main analysis had been set up. Their main concern was that our model did not include random slopes for key within-subjects variables (makeup attractiveness and testosterone). Having looked into this issue over a couple of days, we agreed that not including random slopes typically increases false positive rates and that in the case of our study, the key effect for our interpretation was no longer significant. To minimize misleading other researchers, we contacted the journal immediately and asked to retract the article. Although this was clearly an unfortunate situation, it highlights the importance of open data and analysis code for allowing mistakes to be quickly recognized and the scientific record corrected accordingly.

### Statement on Heyman et al. (2015) by Tom Heyman

The goal of the study was to assess whether the processes that presumably underlie semantic priming effects are automatic in the sense that they are capacity free. For instance, one of the most well-known mechanisms is spreading activation, which entails that the prime (e.g., cat) preactivates related concepts (e.g., dog), thus resulting in a head start. To disentangle prospective processes—those initiated on presentation of the prime, such as spreading activation—from retrospective processes—those initiated on presentation of the target—three different types of stimuli were selected. On the basis of previously gathered word association data, we used symmetrically associated word pairs (e.g., cat–dog; both prime and target elicit one another) as well as asymmetrically associated pairs in the forward direction (e.g., panda–bear; the prime elicits the target but not vice versa) and in the backward direction (e.g., bear–panda; the target elicits the prime but not vice versa). However, I now believe that this manipulation was not successful in teasing apart prospective and retrospective processes. Critically, the three types of stimuli do not solely differ in terms of their presumed prime–target association. That is, I overlooked a number of confounding variables, for one because a priori matching attempts did not take regression effects into account (for more details, see supplementary statement at https://osf.io/bv48h/). Unfortunately, this undercuts the validity of the study’s central claim.

### Statement on Lucas and Diener (2001) by Richard E. Lucas

The article reported three studies that examined the types of situations that extraverts enjoy. Our goal was to assess whether—as intuition and some models of personality might suggest—extraverts are defined by their enjoyment of social situations or whether extraverts are actually more responsive to the pleasantness of situations regardless of whether these are social. We concluded that extraversion correlated more strongly with ratings of pleasant situations than unpleasant situations but not more strongly with social situations than nonsocial situations once pleasantness was taken into account. There are two primary reasons why I have lost confidence in this result. First, the sample sizes are simply too small for the effect sizes one should expect (Schönbrodt & Perugini, 2013). I do not remember how our sample size decisions were made, and the sample sizes vary substantially across studies even though the design was essentially the same. This is especially important given that one important effect from the third and largest study would not have been significant with the sample sizes used in Studies 1 and 2. We did report an internal meta-analysis, but I have become convinced that these procedures cannot correct for other problematic research practices (Vosgerau et al., 2019). Second, many participants were excluded from our final analyses. Two participants were excluded because they were outliers who strongly affected the results. We were transparent about this and reported analyses with and without these outliers. However, the results with the outliers included do not support our hypothesis. We also excluded a second group because their results seemed to indicate that they had misinterpreted the instructions. I still find our explanation compelling, and it may indeed be correct. However, I believe that the appropriate step would be to rerun the study with new procedures that could prevent this misunderstanding. Because we would never have been motivated to look for signs that participants misunderstood the instructions if the results had turned out the way we wanted in the first place, this is an additional researcher degree of freedom that can lead to unreplicable results.

### Statement on Schmukle et al. (2007) by Stefan C. Schmukle

The original main finding was that the implicit gender self-concept measured with the IAT significantly correlated with second-digit/fourth digit (2D:4D) ratios for men (*r* = .36, *p* = .02) but not for women. We used two different versions of a gender IAT in this study (one with pictures and one with words as gender-specific stimuli; *r* = .46), and we had two different 2D:4D measures (the first measure was based on directly measuring the finger lengths using a caliper, and the second was based on measuring the scans of the hands; *r* = .83). The correlation between IAT and 2D:4D was, however, significant only for the combination of picture IAT and 2D:4D scan measure but insignificant for other combinations of IAT and 2D:4D measures. When I was writing the manuscript, I thought that the pattern of results made sense because (a) the research suggested that for an IAT, pictures were better suited as stimuli than words and because (b) I assumed that the scan measures should lead to better results for psychometric reasons (because measurements were averaged across two raters). Accordingly, I reported only the results for the combination of picture IAT and 2D:4D scan measure in the article (for all results, see the long version of the loss-of-confidence statement at https://osf.io/bv48h/). In the meantime, I have lost confidence in this finding, and I now think that the positive association between the gender IAT and 2D:4D is very likely a false-positive result because I should have corrected the *p* value for multiple testing.

### Statement on Silberzahn and Uhlmann (2013) by Raphael Silberzahn and Eric Uhlmann

In 2013, we published an article providing evidence that the meaning of a person’s name might affect the person’s career outcomes. In a large archival data set with more than 200,000 observations, we found that German professionals with noble-sounding last names such as Kaiser (“emperor”), König (“king”), and Fürst (“prince”) were more often found as managers compared with German people with common, ordinary last names such as Koch (“cook”) or Bauer (“farmer”). We applied what we believed to be a solid statistical approach, using generalized estimating equations first, and during the review process applied hierarchical linear modeling and controlled for various potential third variables, including linear controls for name frequency. A postpublication reanalysis by Uri Simonsohn using an expanded version of our data set identified a curvilinear name-frequency confound in the data, whereas we had used only linear controls. Applying the improved matched-names analysis to the larger data set conclusively overturned the original article’s conclusions. Germans with noble and nonnoble names are equally well represented in managerial positions. We subsequently coauthored a collaborative commentary (Silberzahn et al., 2014) reporting the new results. This experience inspired us to pursue our line of work on crowdsourcing data analysis, in which the same data set is distributed to many different analysts to test the same hypothesis and the effect-size estimates are compared (Silberzahn et al., 2018; Silberzahn & Uhlmann, 2015).

### Statement on Smith and Zentall (2016) by Thomas R. Zentall

We have found, paradoxically, that pigeons are indifferent between a signaled 50% reinforcement alternative (leading half of the time to a stimulus that signals 100% reinforcement and otherwise to a stimulus that signals 0% reinforcement) over a guaranteed 100% reinforcement alternative. We concluded that the value of the signal for reinforcement (100% in both cases) determines choice and, curiously, that the signal for the absence of reinforcement has no negative value. More recently, however, using a similar design but involving extended training, we found that there was actually a significant preference for the 50% signaled reinforcement alternative over the 100% reinforcement alternative (Case & Zentall, 2018). This finding required that we acknowledge that there is an additional mechanism involved: the contrast between what was expected and what was obtained (positive contrast). In the case of the 50% reinforcement alternative, 50% reinforcement was expected, but on half of the trials, a signal indicated that 100% reinforcement would be obtained (“elation,” analogous to the emotion felt by a gambler who hits the jackpot). Choice of the 100% reinforcement alternative comes with an expectation of 100% reinforcement, and because 100% reinforcement is obtained, there is no positive contrast and no elation. The recognition of our error in not acknowledging that positive contrast has led to a better understanding of the motivation that gamblers have to gamble in the face of repeated losses and occasional wins.

### Statement on Strand et al. (2018) by Julia Strand

The article reported that when participants listened to spoken words in noise, the cognitive resources necessary to understand the speech (referred to as “listening effort”) were reduced when the speech was accompanied by dynamic visual stimulus—a circle that modulated with the amplitude of the speech. When attempting to replicate and extend that work, I discovered an error in the original stimulus presentation program that was responsible for the observed effect. The listening-effort task we used was based on response time, so the critical comparison was participant response times in conditions with and without the visual stimulus. There was an unintentional delay set in the timer of the condition without the visual stimulus, leading to artificially slowed response times in that condition. We contacted the journal, and they invited us to submit a replacement article. Given that the timing delay affected every observation for one condition in a systematic way, it was straightforward to reanalyze the data and present the results as they would have been without the error. The original article was not retracted but now links to the new article (Strand et al., 2020) that presents the corrected results.

### Statement on Vazire (2010) by Simine Vazire

In this article, I suggested a model in which self-reports are more accurate than peer reports for traits that are low in observability and low in evaluativeness, whereas peer reports are more accurate than self-reports for traits that are high in observability and high in evaluativeness. The main issue was that I ran many more analyses than I reported, and I cherry-picked which results to report. This is basically *p*-hacking, but because most of my results were not statistically significant, I did not quite successfully *p*-hack by the strict definition. Still, I cherry-picked the results that made the contrast between self-accuracy and peer accuracy the most striking and that fit with the story about evaluativeness and observability. That story was created post hoc and chosen after I had seen the pattern of results.

### Statement on Willén and Strömwall (2012) by Rebecca M. Willén

In this study, I evaluated the criteria used by Swedish courts for assessing credibility of plaintiffs’ accounts. The main reasons for my loss of confidence in the results reported are listed below.

First, the main coder (myself) was not blind to the veracity of the statements. In addition, the main coder had also conducted the interviews, which means that she might have been influenced by the memory of nonverbal cues that were not supposed to have influenced the codings. The second coder was blind and did indeed come to different conclusions in his codings. These differences may have been a consequence of the conditions and nonverbal cues being known to the main coder, and this possibility remained undisclosed in the article.

Second, all four hypotheses described as confirmatory in the introduction of the article were in fact not formalized until after the data had been collected. It could be argued that the first three hypotheses were “obvious” and thereby implicitly already decided on. The fourth hypothesis, however, was far from obvious, and it was the result of exploratory analyses made by myself.

Finally, no gender differences were predicted, and gender was never planned to be analyzed at all. The gender findings are thus the result of exploratory analyses. This fact is, however, never made very explicit; instead, these unexpected results are highlighted even in the abstract.

That said, I do think there is reason to believe that one particular main finding is worth trying to replicate: “False and truthful confessions by 30 offenders were analyzed, and few significant effects were obtained.” That is, true and false statements by criminally experienced offenders might be more difficult to distinguish than true and false statements provided by the typical participants in deception and interrogation research (i.e., undergraduates without criminal experience).

### Statement on Witt and Proffitt (2008) by Jessica K. Witt

The article reported that squeezing a rubber ball interferes with the processes necessary for the perceiver’s ability to reach to a target to affect perceived distance to the target (Experiment 3a). Participants judged the distance to targets that were beyond the reach of the arm, then picked up a conductor’s baton and reached to them. One group of participants applied a constant, firm pressure on a rubber ball while making their distance judgments, and another group did not. There are two primary flaws that cast doubt on the findings. One concerns the methodology. The sample sizes were small, so statistical power was likely to be quite low. The other concern regards the statistical analysis. The analysis reported in the article used an incorrectly specified model. Specifically, we calculated the mean estimated distance for each participant at each distance for a total of 10 estimates per participant, then analyzed these means as if they were independent observations. This inflated the degrees of freedom, which resulted in lower *p* values. When the data are analyzed correctly, the statistical significance of the critical effect of ball squeeze on estimated distance depends on whether or not an outlier is removed (for full results, see long version of the loss-of-confidence statement at https://osf.io/bv48h/). Model misspecification and low sample sizes also applied to Experiments 1, 2, and 3b. For Experiment 1, when the data are analyzed correctly, statistical significance depends on the exclusion of two outliers. For Experiment 2, the critical effect of tool condition was not significant; no outliers were identified. There were only 4 participants per condition, making the experimental outcomes inconclusive. For Experiment 3b, the article originally reported a null effect; upon reanalysis, the effect was still null. Experiment 4 is believed to have been analyzed correctly on the basis of the reported degrees of freedom, but those data have been lost and therefore cannot be confirmed. With such low statistical power, little confidence can be had that the reported data support the idea that squeezing a ball can interfere with the effect of tool use on estimated distance.

### Statement on Yarkoni et al. (2005) by Tal Yarkoni

This study used a dynamic decision-making task to investigate the neural correlates of temporally extended decision-making. The central claim was that activation in areas of right lateral prefrontal cortex strongly and selectively predicted choice behavior in two different conditions; peak between-subjects brain-behavior correlations were around *r* = .75. I now think most of the conclusions drawn in this article were absurd on their face. My understanding of statistics has improved a bit since writing the article, and it is now abundantly clear to me that (a) I *p*-hacked to a considerable degree (e.g., the choice of cluster thresholds was essentially arbitrary) and that (b) because of the “winner’s curse,” statistically significant effect sizes from underpowered studies cannot be taken at face value (see Yarkoni, 2009). Beyond these methodological problems, I also now think the kinds of theoretical explanations I proposed in the article were ludicrous in their simplicity and naivete—so the results would have told us essentially nothing even if they were statistically sound (see Meehl, 1967, 1990).

## Discussion of the Loss-of-Confidence Statements

The studies for which we received statements spanned a wide range of psychological domains (stereotypes, working memory, auditory perception, visual cognition, face perception, personality and well-being, biologically driven individual differences, social cognition, decision-making in nonhuman animals, deception detection) and employed a diverse range of methods (cognitive tasks, implicit and explicit individual differences measures, archival data analyses, semistructured interviews, functional MRI), demonstrating the broad relevance of our project. Overall, the respective original articles had been cited 1,559 times as of April 27, 2020, according to Google Scholar, but the number of citations varied widely, from nine to 740. The reasons given for the submitters’ loss of confidence also varied widely, with some statements providing multiple reasons. Broadly speaking, however, we can group the explanations into three general categories.

### Methodological error

Five of the statements reported methodological errors in the broadest sense. In three instances, submitters (Jones & DeBruine; Silberzahn & Uhlmann; Witt) lost confidence in their findings upon realizing that their key results stemmed from misspecified statistical models. In those three cases, the submitters discovered, after publication, that a more appropriate model specification resulted in the key effect becoming statistically nonsignificant. In another instance, Carlsson reported that upon reconsideration, two studies included in his article actually tested different hypotheses—a reanalysis testing the same hypotheses in Study 2 actually failed to fully support the findings from Study 1. Finally, Strand lost confidence when she found out that a programming error invalidated her findings.

### Invalid inference

Four of the statements reported invalid inferences in the broadest sense. In two cases (Heyman and Yarkoni), the submitters attributed their loss of confidence to problems of validity—that is, to a discrepancy between what the reported results actually showed (a statistically significant effect of some manipulation or measure) and what the article claimed to show (a general relationship between two latent constructs). In a similar vein, Zentall lost confidence in a conclusion when a follow-up experiment revealed that an extension of the experimental procedures suggested that the original mechanism was not sufficient to account for the phenomenon. Although the latter loss-of-confidence statement might be closest to normative assumptions about how science advances—new empirical insights lead to a revision of past conclusions—it also raises interesting questions: At what point should researchers lose confidence in a methodological decision made in one study based on the results of other studies that are, in principle, also fallible?

### *p*-hacking

Seven of the statements (Carlsson, Chabris, Lucas, Yarkoni, Schmukle, Vazire, and Willén) reported some form of *p*-hacking—specifically, failing to properly account for researcher degrees of freedom when conducting or reporting the analyses. We hasten to emphasize that our usage of “*p*-hacking” here does not imply any willful attempt to mislead. Indeed, some of the submitters noted that the problems in question stemmed from their poor (at the time) understanding of relevant statistical considerations. The statement by Lucas also highlights how subtle researcher degrees of freedom can affect analyses: Although the justification for a specific exclusion criterion still seems compelling, the researcher would not have been motivated to double-check data points if the desired results had emerged in the initial analysis.

## Results and Discussion of the Anonymous Online Survey

**Fig. 1.**
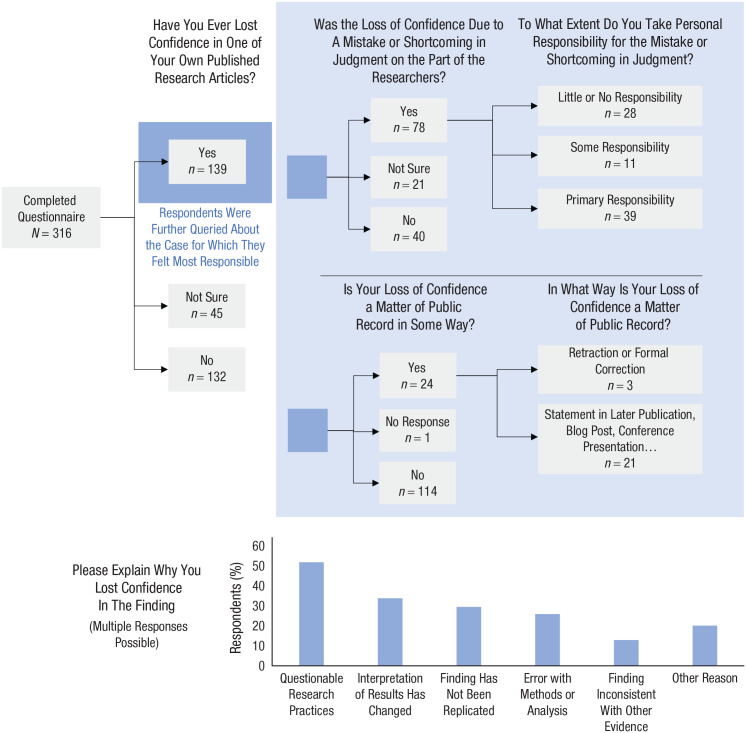
An overview of the findings from the loss-of-confidence survey.

Overall, 316 scientists completed the survey. Most (93%) reported being affiliated with a university or a research institute, and all career stages from graduate students to tenured professors were represented. We did not limit the survey to particular fields of research but asked respondents to indicate their department (if applicable); 43% did not report a department, 37% worked at a psychology department, and the remaining respondents were distributed over a broad range of fields (e.g., business, economics, medicine). Almost all respondents reported working either in Europe (44%) or the United States (47%). Figure 1 provides an overview of the survey results.

Almost half of the respondents (44%) reported losing confidence in at least one of their findings. Another 14% were not sure whether they had lost confidence according to our definition for a variety of reasons. For example, some reported that their confidence in one of their own research articles was low to begin with; some had lost confidence in their theoretical explanation but not in the general effect—or conversely, in the effect but not in the theory; others doubted whether their results would generalize to other contexts. Respondents who reported losing confidence were then asked to elaborate on the case for which they felt most responsible.^4^ Of the respondents who stated that they had experienced a loss of confidence, more than half (56%) said that it was due to a mistake or shortcoming in judgment on the part of the researchers, and roughly one in four (28%) took primary responsibility for the error.

Strikingly, the primary reason indicated for a loss of confidence was self-admitted questionable research practices (e.g., *p*-hacking and selective reporting; 52%). However, a broad variety of other reasons were also reported. The loss of confidence was a matter of public record in fewer than a fifth of the reported cases (17%), and if it was a matter of public record, the outlets primarily chosen (statement in later publication, conference presentation, social media posting) were not directly linked to the original research article. Respondents whose loss of confidence was not public reported multiple reasons for the lack of disclosure. Many felt insufficiently sure about the loss of confidence to proceed (68%). Some stated the belief that public disclosure was unnecessary because the finding had not attracted much attention (46%), expressed concerns about hurting the feelings of coauthors (33%), or cited the lack of an appropriate venue (25%), uncertainty about how to best communicate the matter (25%), and worries about how the loss of confidence would be perceived (24%).

On the whole, these survey results suggest a nuanced view of losses of confidence. Researchers may start to question their own findings for a broad variety of reasons, and different factors may then keep them from publicly disclosing this information. Collectively, the responses suggest that a sizeable proportion of active researchers has lost confidence in at least one of their findings—often because of a recognized error of their own commission.

Note that our respondents do not constitute a representative sample of researchers. Furthermore, estimating article-level rather than researcher-level loss of confidence requires assumptions and extrapolations.^5^ Thus, caution should be exercised when interpreting the specific numerical estimates reported here. Nevertheless, one can attempt a very conservative extrapolation: More than 1 million academic articles are currently published each year (Jinha, 2010). Supposing that at least a third of these are empirical research reports, and that even just 1% of these reports are affected, that still leaves thousands of articles published each year that will eventually lose the confidence of at least some of their authors—often because of known errors yet typically without any public disclosure.

## General Discussion

The Loss-of-Confidence Project raises a number of questions about how one should interpret individual self-corrections.

First, on a substantive level, how should one think about published empirical studies in cases in which the authors have explicitly expressed a loss of confidence in the results? One intuitive view is that authors have no privileged authority over “their” findings, and thus such statements should have no material impact on a reader’s evaluation. On the other hand, even if authors lack any privileged authority over findings they initially reported, they clearly often have privileged access to relevant information. This is particularly salient for the *p*-hacking disclosures reported in the loss-of-confidence statements. Absent explicit statements of this kind, readers would most likely not be able to definitively identify the stated problems in the original report. In such cases, we think it is appropriate for readers to update their evaluations of the reported results to accommodate the new information.

Even in cases in which a disclosure contributes no new methodological information, one might argue that the mere act of self-correction should be accorded a certain weight. Authors have presumably given greater thought to and are more aware of their own study’s potential problems and implications than a casual reader. The original authors may also be particularly biased to evaluate their own studies favorably—so if they have nonetheless lost confidence, this might heuristically suggest that the evidence against the original finding is particularly compelling.

Second, on a metalevel, how should one think about the reception one’s project received? On the one hand, one could argue that the response was about as positive as could reasonably be expected. Given the unconventional nature of the project and the potentially high perceived cost of public self-correction, the project organizers (J. M. Rohrer, C. F. Chabris, T. Yarkoni) were initially unsure whether the project would receive any submissions. From this perspective, even the 13 submissions we ultimately received could be considered a clear success and a testament to the current introspective and self-critical climate in psychology.

On the other hand, the survey responses we received suggest that the kinds of errors disclosed in the statements are not rare. Approximately 12% of the 316 survey respondents reported losing confidence in at least one of their articles for reasons that matched our stringent submission criteria (i.e., because of mistakes that the respondent took personal responsibility for), and nearly half acknowledged a loss of confidence more generally.

This suggests that potentially hundreds, if not thousands, of researchers could have submitted loss-of-confidence statements but did not do so. There are many plausible reasons for this, including not having heard of the project. However, we think that at least partially, the small number of submitted statements points to a gap between researchers’ ideals and their actual behavior—that is, public self-correction is desirable in the abstract but difficult in practice.

### Fostering a culture of self-correction

As has been seen, researchers report a variety of reasons for both their losses of confidence and their hesitation to publicly disclose a change in thinking. However, we suggest that there is a broader underlying factor: In the current research environment, self-correction, or even just critical reconsideration of one’s past work, is often disincentivized professionally. The opportunity costs of a self-correction are high; time spent on correcting past mistakes and missteps is time that cannot be spent on new research efforts, and the resulting self-correction is less likely to be judged a genuine scientific contribution. Moreover, researchers may worry about self-correction potentially backfiring. Corrections that focus on specific elements from an earlier study might be perceived as undermining the value of the study as a whole, including parts that are in fact unaffected by the error. Researchers might also fear that a self-correction that exposes flaws in their work will damage their reputation and perhaps even undermine the credibility of their research record as a whole.

To tackle these obstacles to self-correction, changes to the research culture are necessary. Scientists make errors (and this statement is certainly not limited to psychological researchers; see e.g., Eisenman et al., 2014; García-Berthou & Alcaraz, 2004; Salter et al., 2014; Westra et al., 2011), and rectifying these errors is a genuine scientific contribution—whether it is done by a third party or the original authors. Scientific societies could consider whether they want to more formally acknowledge efforts by authors to correct their own work. Confronted with researchers who publicly admit to errors, other researchers should keep in mind that willingness to admit error is not a reliable indicator of propensity to commit errors—after all, errors are frequent throughout the scientific record. On the contrary, given the potential (or perceived) costs of individual self-corrections, public admission of error could be taken as a credible signal that the issuer values the correctness of the scientific record. However, ultimately, given the ubiquity of mistakes, we believe that individual self-corrections should become a routine part of science rather than an extraordinary occurrence.

### Different media for self-correction

Unfortunately, good intentions are not enough. Even when researchers are committed to public self-correction, it is often far from obvious how to proceed. Sometimes, self-correction is hindered by the inertia of journals and publishers. For example, a recent study suggested that many medical journals published correction letters only after a significant delay, if at all (Goldacre et al., 2019), and authors who tried to retract or correct their own articles after publication have encountered delays and reluctance from journals (e.g., Grens, 2015). Even without such obstacles, there is presently no standardized protocol describing what steps should be taken when a loss of confidence has occurred.

Among the participants of the Loss-of-Confidence Project, Fisher et al. (2015) decided to retract their article after they became aware of their misspecified model. But researchers may often be reluctant to initiate a retraction given that retractions occur most commonly as a result of scientific misconduct (Fang et al., 2012) and are therefore often associated in the public imagination with cases of deliberate fraud. To prevent this unwelcome conflation and encourage more frequent disclosure of errors, journals could introduce a new label for retractions initiated by the original authors (e.g., “Authorial Expression of Concern” or “voluntary withdrawal”; see Alberts et al., 2015). Furthermore, an option for authorial amendments beyond simple corrections (up to and including formal versioning of published articles) could be helpful.

Thus, it is not at all clear that widespread adoption of retractions would be an effective, fair, or appropriate approach. Willén (2018) argued that retraction of articles in which questionable research practices (QRPs) were employed could deter researchers from being honest about their past actions. Furthermore, retracting articles because of QRPs known to be widespread (e.g., John et al., 2012) could have the unintended side effect that some researchers might naively conclude that a lack of a retraction implies a lack of QRPs. Hence, Willén suggested that all articles should be supplemented by transparent retroactive disclosure statements. In this manner, the historical research record remains intact because information would be added rather than removed.

Preprint servers (e.g., PsyArXiv.com) and other online repositories already enable authors to easily disclose additional information to supplement their published articles or express their doubts. However, such information also needs to be discoverable. Established databases such as PubMed could add links to any relevant additional information provided by the authors. Curate Science (curatescience.org), a new online platform dedicated to increasing the transparency of science, is currently implementing retroactive statements that could allow researchers to disclose additional information (e.g., additional outcome measures or experimental manipulations not reported in the original article) in a straightforward, structured manner.

Another, more radical step would be to move scientific publication entirely online and make articles dynamic rather than static such that they can be updated on the basis of new evidence (with the previous version being archived) without any need for retraction (Nosek & Bar-Anan, 2012). For example, the *Living Reviews* journal series in physics by Springer Nature allows authors to update review articles to incorporate new developments.

The right course of action once one has decided to self-correct will necessarily depend on the specifics of the situation, such as the reason for the loss of confidence, publication norms that can vary between research fields and evolve over time, and the position that the finding takes within the wider research. For example, a simple but consequential computational error may warrant a full retraction, whereas a more complex confound may warrant a more extensive commentary. In research fields in which the published record is perceived as more definitive, a retraction may be more appropriate than in research fields in which published findings have a more tentative status. In addition, an error in an article that plays a rather minor role in the context of the wider research may be sufficiently addressed in a corrigendum, whereas an error in a highly cited study may require a more visible medium for the self-correction to reach all relevant actors.

That said, we think that both the scientific community and the broader public would profit if additional details about the study, or the author’s reassessment of it, were always made public and always closely linked to the original article—ideally in databases and search results as well as the publisher’s website and archival copies. A cautionary tale illustrates the need for such a system: In January 2018, a major German national weekly newspaper published an article (Kara, 2018a) that uncritically cited the findings of Silberzahn and Uhlmann (2013). Once the journalist had been alerted that these findings had been corrected in Silberzahn et al. (2014), she wrote a correction to her newspaper article that was published within less than a month of the previous article (Kara, 2018b), demonstrating swift journalistic self-correction and making a strong point that any postpublication update to a scientific article should be made clearly visible to all readers of the original article.

## Outlook

All of these measures *could* help to transform the cultural norms of the scientific community, bringing it closer to the ideal of self-correction. Naturally, it is hard to predict which ones will prove particularly fruitful, and changing the norms of any community is a nontrivial endeavor. However, it might be encouraging to recall that over the past few years, scientific practices in psychology have already changed dramatically (Nelson et al., 2018). Hence, a shift toward a culture of self-correction may not be completely unrealistic, and psychology, with its increasing focus on openness, may even serve as a role model for other fields of research to transform their practices.

Finally, it is quite possible that fears about negative reputational consequences are exaggerated. It is unclear whether and to what extent self-retractions actually damage researchers’ reputations (Bishop, 2018). Recent acts of self-correction such as those by Carney (2016), which inspired our efforts in this project, Silberzahn and Uhlmann (Silberzahn et al., 2014), Inzlicht (2016), Willén (2018), and Gervais (2017) have received positive reactions from within the psychological community. They remind us that science can advance at a faster pace than one funeral at a time.

## Transparency

*Action Editor:* Chris Crandall

*Editor:* Laura A. King

## Notes

1. Guidelines to promote openness (e.g., Nosek et al., 2015) might partly reduce this asymmetry and thus make it easier for third parties to spot flaws.

2. An archived version of the website can be found at https://web.archive.org/web/20171212055615/https://lossofconfidence.com/.

3. Readers are cautioned to infer nothing about original authors who did not join or sign a loss-of-confidence statement about their own articles. In some cases, these authors approved of the submission but did not get involved otherwise; in others, they had already left the field of research.

4. Respondents who were not sure whether they had experienced a loss of confidence could also answer the follow-up questions. However, many decided not to answer, and for those who answered, responses are hard to interpret given the broad variety of scenarios they were referring to. Thus, we decided to restrict the following analyses to respondents with an unambiguous loss of confidence.

5. In the survey, we also asked researchers to indicate in how many of their articles they had lost confidence. An analysis of these numbers suggested that respondents had collectively lost confidence in more than 10% of their publications in total or more than 7% counting only those articles in which they had lost confidence because of an error for which they took primary responsibility. Of course, these are extrapolations based on retrospective self-reports, and we cannot assume respondents can give perfect estimates of the relevant quantities. For this reason, a number of our key analyses focus on the respondents’ description of the one case for which they felt most responsible.
